# Anatomical Study of the Duodenojejunal Uncinate Process Vein: A Key Landmark for Mesopancreatoduodenal Resection During Pancreaticoduodenectomy

**DOI:** 10.1002/ags3.12518

**Published:** 2021-10-12

**Authors:** Masahiko Honjo, Taiji Tohyama, Kohei Ogawa, Kei Tamura, Katsunori Sakamoto, Akihiro Takai, Jota Watanabe, Hiromi Ohtani, Yasutsugu Takada

**Affiliations:** ^1^ Department of Hepato‐Biliary‐Pancreatic and Breast Surgery Ehime University Graduate School of Medicine Shitsukawa, Toon Japan; ^2^ Department of Surgery Ehime Prefectural Central Hospital Matsuyama Japan; ^3^ Department of Surgery Kurashiki Medical Center Kurashiki Japan

**Keywords:** duodenojejunal uncinate process vein, jejunal vein, mesopancreas, mesopancreatoduodenum, pancreaticoduodenectomy

## Abstract

**Background:**

The mesopancreas or mesopancreatoduodenum is an important anatomical concept during pancreaticoduodenectomy (PD) in patients with periampullary carcinoma. This study investigated whether the duodenojejunal uncinate process vein (DJUV), which is defined as the vein draining from the upper jejunum to the superior mesenteric vein adjacent to the uncinate process, is a useful anatomical landmark for the caudal border of mesopancreatoduodenum resection during PD.

**Methods:**

This study enrolled 100 adult patients with hepatobiliary pancreatic disease who underwent preoperative multidetector‐computed tomography (CT). The anatomy of the key blood vessels involved during PD, and the relationship between these vessels and the DJUV, were analyzed by preoperative CT.

**Results:**

The first jejunal vein was the DJUV in 85 cases, whereas the second jejunal vein was the DJUV in 15 cases. Furthermore, the DJUV was classified into two subtypes depending on its positional relationship with the superior mesenteric artery (SMA). The inferior pancreaticoduodenal artery and vein were located on the cranial side of the DJUV in all cases. The distance between the middle colonic artery, used as a guide for regional lymph nodes, and the point where the DJUV intersected the SMA was within 10 mm in 80% of cases. These results imply that using the DJUV as a landmark for the caudal border of the mesopancreatoduodenum provides a safe approach and enables sufficient dissection of regional lymph nodes and tissues around the SMA.

**Conclusion:**

The DJUV may be a useful anatomical landmark for the caudal border of the mesopancreatoduodenum resection during PD.

## Graphical abstract

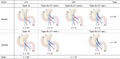

## 1 INTRODUCTION

Multimodal treatment strategies have gradually improved the prognosis of resectable pancreatic cancer, but it remains poor, with a 5‐y survival rate of 20%–25% after surgery.^1^, ^2^, ^3^, ^4^ Pancreaticoduodenectomy (PD) is the only potential curative treatment and is recognized as one of the most influential determinants of survival.^1^, ^5^ In 2007, Gockel et al proposed the concept of the mesopancreas, defined as a firm and well‐vascularized structure that extends from the posterior face of the head of the pancreas to behind the superior mesenteric vein (SMV) and superior mesenteric artery (SMA).^6^ It consists of areolar tissue, adipose tissue, blood vessels, peripheral nerves, and lymph nodes.^6^, ^7^ This area is recognized as the primary positive resection margin site and the main site of local recurrence.^8^

The effectiveness of resection of the mesopancreas or pancreato‐duodenojejunal mesentery, ie, mesopancreatoduodenum, has been reported.^7^, ^9^, ^10^, ^11^ However, the standard procedure for mesopancreas or mesopancreatoduodenum resection has not been established because the resection boundaries remain unclear.^9^, ^12^ Anatomical landmarks that can be identified during surgery include anatomical membranes and blood vessels. The hepatoduodenal ligament, the common hepatic artery, and the root of the SMA and celiac artery are present on the cranial side of the pancreas, and the anterior renal fascia on the inferior vena cava, renal vein, and aorta are present on the dorsal side.^13^ Therefore, the boundaries are relatively clear at these sites. However, the caudal borders, particularly the mesenteric dissecting line, are confusing and vary among reports.^10^, ^11^, ^14^, ^15^, ^16^

The purpose of this study was to investigate whether the pancreato‐duodenojejunal mesenteric veins draining into the SMV at the level of the uncinate process, which we named the “duodenojejunal uncinate process vein (DJUV)” could be used as a caudal anatomical landmark for mesopancreas or mesopancreatoduodenum resection during PD. Specifically, we analyzed the anatomy of the key blood vessels involved in PD, and assessed the relationship between these vessels and the DJUV using three‐dimensional computed tomography (3D‐CT) images based on thin‐slice CT examinations.

## 2 PATIENTS AND METHODS

### 2.1 Patients

All procedures involving human participants followed the ethical standards of the institution and research committee and with the 1964 Declaration of Helsinki and its later amendments or comparable ethical standards. This study was approved by the Ethics Review Boards of Ehime University School of Medicine and Ehime Central Hospital.

In total, 100 patients with hepatobiliary pancreatic disease who underwent preoperative multidetector CT at Ehime University or Ehime Central Hospital between April 2016 and May 2020 were included. The CT data were retrospectively reviewed. We enrolled 68 male and 32 female patients of median age 65 y (range 20–86 y). Their primary conditions were hepatocellular carcinomas (n = 25), metastatic liver tumors (n = 9), liver transplant donor status (n = 11), hilar cholangiocarcinomas (n = 5), distal bile duct carcinomas (n = 16), duodenal papillary tumors (n = 2), a duodenal gastrointestinal stromal tumor (n = 1), gallbladder cancers (n = 2), and pancreatic tumors that did not affect the duodenomesopancreatic region (n = 29; pancreatic body and tail carcinomas [n = 14], intraductal papillary mucinous neoplasms [n = 7], neuroendocrine tumors [n = 5], a serous cystic neoplasm [n = 1], a solid pseudopapillary tumor [n = 1], and a lymphoepithelial cyst [n = 1]). Patients with pancreatic head cancers and large cystic tumors of the pancreas head were excluded, given the hemodynamic changes in the pancreatic head caused by vascular invasion and/or tumor expansion. Also, portal hypertension patients with CT‐revealed varices around the lower esophagus or stomach, or with submucosal varices apparent on endoscopic images, were excluded.

### 2.2 Definition of DJUV

The following conditions were required for the caudal border of the mesopancreatoduodenum resection for PD. The excisional area included all blood vessels, the nerve plexus, and regional lymph nodes that should be resected. It should be a macroscopically recognizable anatomical structure during surgery. We have recently focused on the jejunal vein in the pancreato‐duodenojejunal mesentery that drains from the upper jejunum to the SMV adjacent to the uncinate process via the third and fourth portions of the duodenum. We named this vein the DJUV, and hypothesized that it may be a candidate for the caudal border of the mesopancreatoduodenum during resection.

### 2.3 CT protocol and 3D reconstruction

All CT examinations were performed using a 256‐slice CT scanner (Brilliance iCT, Philips Healthcare, Andover, MA) running a standardized, multiphasic hepatobiliary‐pancreatic protocol. The tube voltage was 120 kVp and the “automated tube current modulation” feature was employed in all cases. The other parameters were as follows: detector collimation, 128 × 0.625 mm; field‐of‐view (FOV), 350 mm; rotation time, 0.4 sec; and spiral pitch, 0.446. If dynamic CT was planned, precontrast images were obtained first. All patients were given nonionic, iodinated contrast (300–370 mgI/mL) via a 20G intravenous catheter in the antecubital vein; we employed a power injector. The dose of contrast material was determined based on body surface area derived using the DuBois formula: body surface area (m^2^) = 0.007184 × height (cm) 0.725 × weight (kg) 0.425. All patients were given 22 gI/m^2^ of contrast material over 30 sec. This injection technique was based on a bolus‐tracking method. Arterial phase imaging commenced 15 sec later, by which time the trigger threshold of 150 HU in the abdominal aorta had been attained. The portal‐venous and delayed phases commenced at 40 and 100 sec after attainment of the trigger threshold, respectively. All series were reconstructed using an iterative reconstruction algorithm (iDose 4; Philips Healthcare).^17^

**FIGURE 1:**
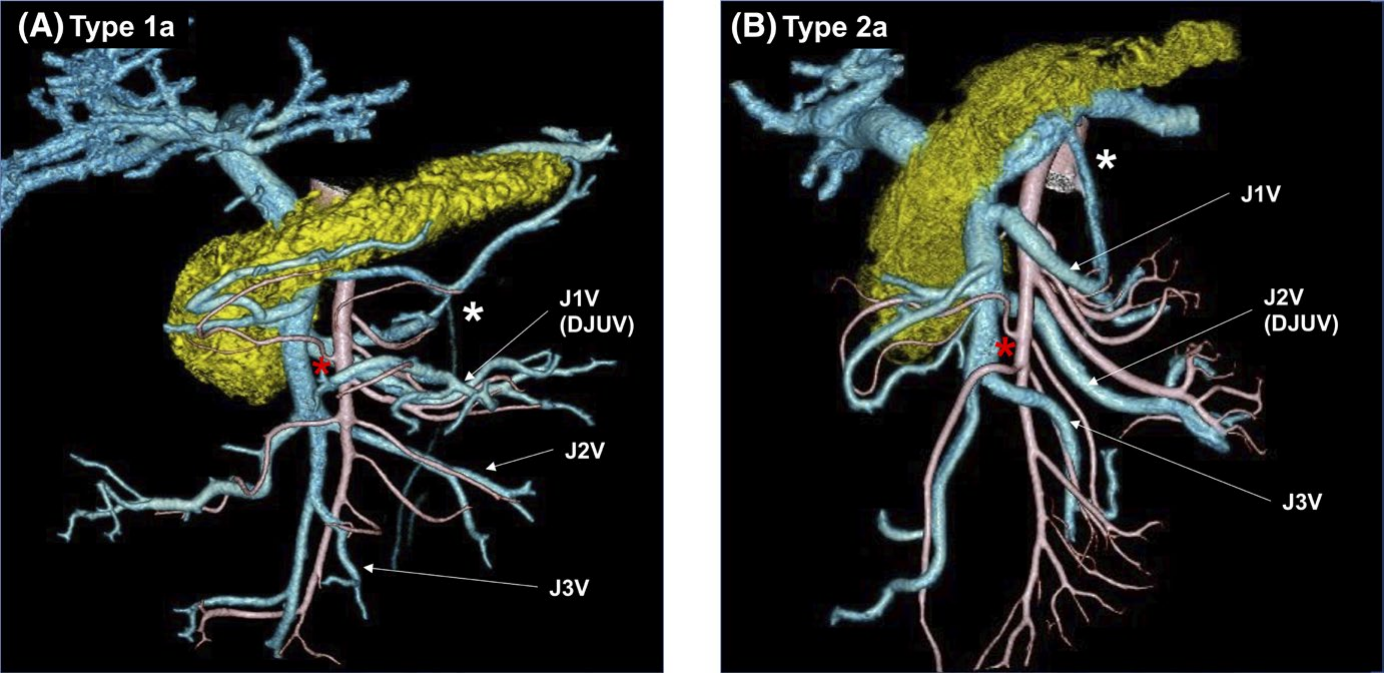
Typical DJUV images. (A) Type 1a, in which the J1V corresponds to the DJUV, and runs dorsal to the SMA. (B) Type 2a, in which the J2V corresponds to the DJUV, and runs dorsal to the SMA. DJUV, duodenojejunal uncinate process vein; J1‐4A, first to fourth jejunal arteries; J1V‐J3V, first to third jejunal veins; white asterisk, inferior mesenteric vein; red asterisk, middle colic artery

Synapse Vincent 3D software (Fujifilm, Tokyo, Japan) was used to generate the 3D‐CT images. Pancreatic 3D images and arteriographs were obtained during the arterial phase and fused to yield portographs with 0.5‐mm‐thick slices. All 3D‐CT images were reconstructed by a single surgeon. The tributaries of the SMV were numbered in order from the confluence of the splenic vein to distal of the SMV, such as the first and second jejunal veins (J1V and J2V, respectively), and the jejunal artery branches were numbered from the root of the SMA (Figure 1).

### 2.4 Anatomical evaluation

The following anatomical findings were analyzed: which jejunal vein corresponded to the DJUV; anatomical variations in the inferior pancreaticoduodenal veins (IPDVs) and inferior pancreaticoduodenal artery (IPDA), and their positional relationships with the DJUV were examined; the distance between the bifurcation of the middle colonic artery (MCA) and the intersection of the DJUV and SMA (the DJUV intersecting point) was measured; and the number of jejunal arteries branching between the root of the SMA and the DJUV intersecting point were counted.

## 3 RESULTS

### 3.1 Jejunal veins corresponding to the DJUV

**FIGURE 2:**
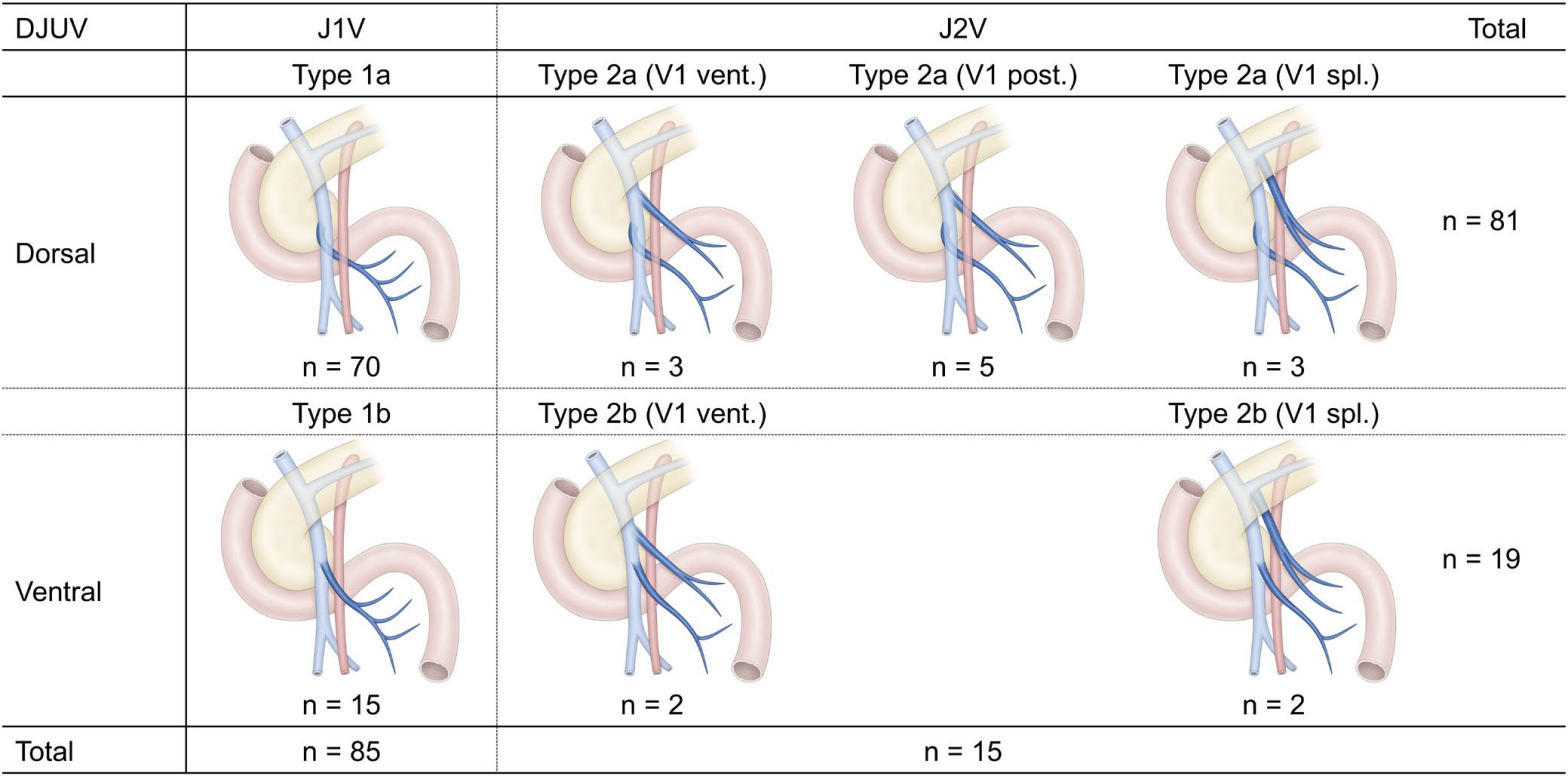
Anatomical variations in the DJUV. The J1V was the DJUV (Type 1) in 85 cases, whereas the J2V was the DJUV in 15 cases (Type 2). Next, the DJUV was divided into two subtypes depending on its positional relationship with the SMA, ie, running dorsal (subtype a) or ventral (subtype b) to the SMA. There were 70 cases of Type 1a, 15 cases of Type 1b, 11 cases of Type 2a, and four cases of Type 2b. In Type 2a, the J1V ran along the ventral side of the SMA in three cases, along the dorsal side of the SMA in five cases, and the J1V drained into the splenic vein in three cases. In Type 2b, the J1V ran along the ventral side of the SMA in two cases, and the J1V drained into the splenic vein in two cases. SMA, superior mesenteric artery; DJUV, duodenojejunal uncinate process vein; J1V first jejunal vein; J2V second jejunal vein; V1 vent., the first jejunal vein running ventral to the SMA; V1 dor., the first jejunal vein running dorsally to the SMA; V1 spl., first jejunal vein drained into the splenoportal confluence

Either the J1V or the J2V corresponded to the DJUV in all cases. The J1V was the DJUV (Type 1) in 85 cases, whereas the J2V was the DJUV (Type 2) in 15 cases (Figure 2). The DJUV was divided into two subtypes, depending on its positional relationship with the SMA, ie, running dorsal (subtype a) or ventral (subtype b) to the SMA. There were 70 cases of Type 1a, 15 of Type 1b, 11 of Type 2a, and four of Type 2b. Among the Type 2a and Type 2b cases, the J1V drained into the splenoportal confluence (splenic vein near the confluence of the SMV) in three and two cases, respectively.

### 3.2 Anatomical variations in the IPDV

**FIGURE 3:**
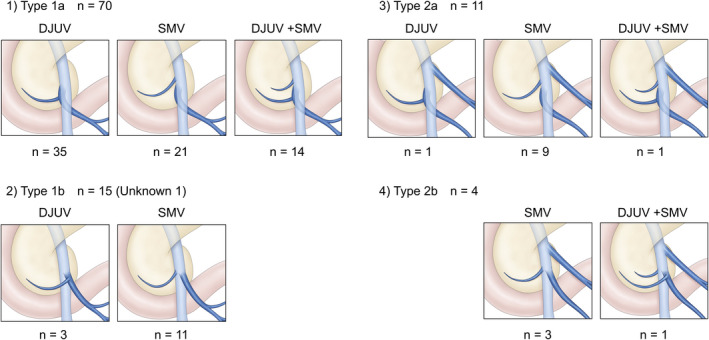
Anatomical variations in the IPDV. Three variations were identified: blood return to the DJUV only, blood return to the SMV only, and blood return to both. (1) Type 1a included 35, 21, and 14 cases, respectively. (2) Type 1b included 3, 11, and 0 cases, respectively. (3) Type 2a included 1, 9, and 1 cases, respectively. (4) Type 2b included 0, 3, and 1 cases, respectively. IPDV, inferior pancreaticoduodenal vein; DJUV, duodenojejunal uncinate process vein; SMV, superior mesenteric vein

As the IPDVs could not be visualized in one case, 99 cases were analyzed. Three variations were identified: the IPDV drained into the DJUV only, into the SMV only, or into both (Figure 3). Variations were observed in 35, 21, and 14 Type 1a patients; 3, 11, and 0 Type 1b patients; 1, 9, and 1 Type 2a patients; and 0, 3, and 1 Type 2b patients, respectively. In no case did the IPDV join the SMV distal to the DJUV.

### 3.3 Anatomical variations in the IPDA

**FIGURE 4:**
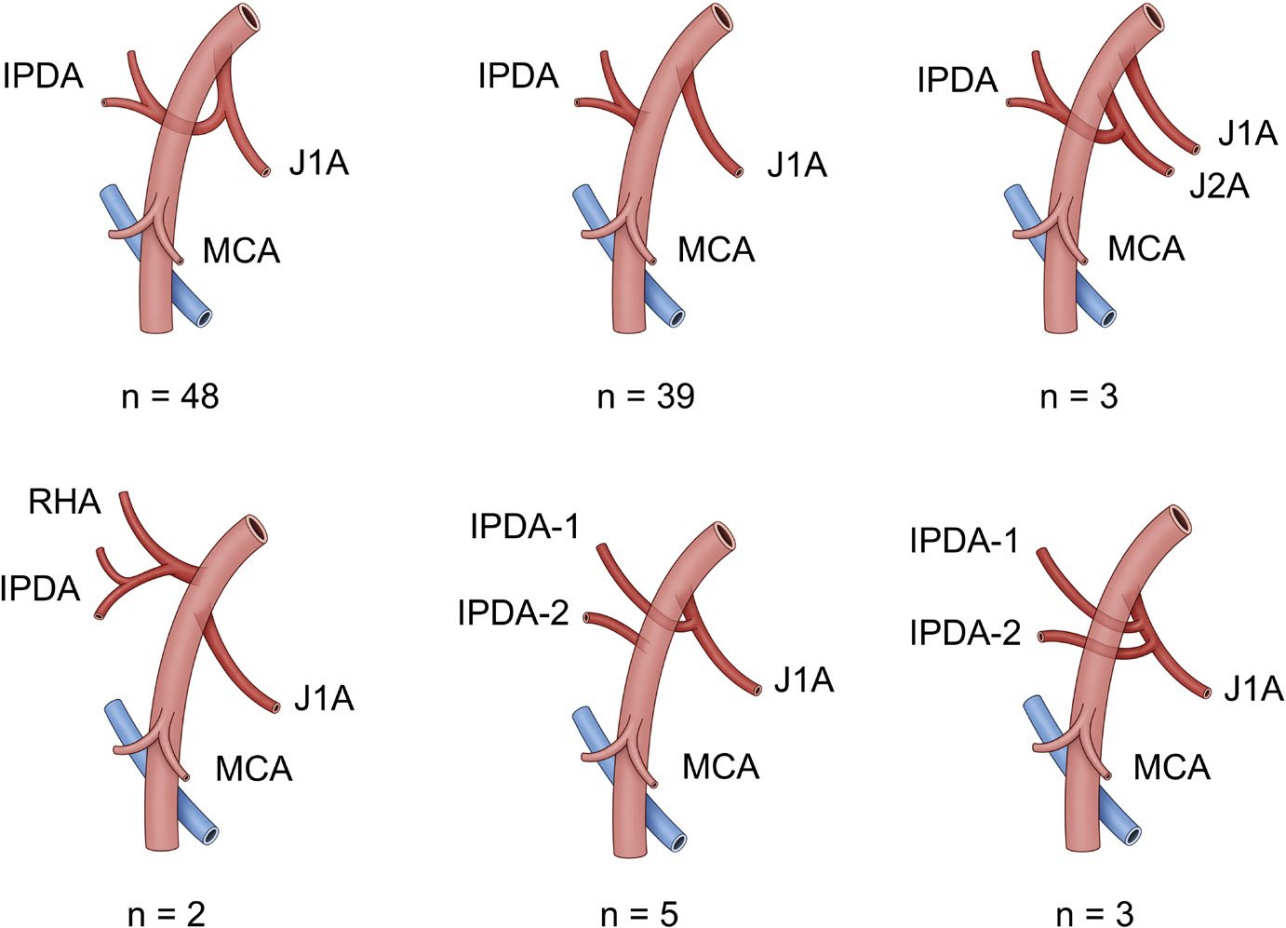
Anatomical variations in the IPDA. A single IPDA branched from the J1A in 48 cases, branched directly from the SMA in 39 cases, branched from the J2A in three cases, and branched from a replaced right hepatic artery in two cases. Two IPDAs were observed in the remaining eight cases: one from the J1A and the other from the SMA (n = 5), or both from the J1A (n = 3). IPDA, inferior pancreatoduodenal artery; J1A, first jejunal artery; J2A, second jejunal artery; RHA, right hepatic artery; MCA, middle colic artery; DJUV, duodenojejunal uncinate process vein

Only one IPDA was confirmed in most cases (Figure 4). The IPDA branched from the J1A in 48 cases, branched directly from the SMA in 39 cases, branched from the J2A in three cases, and branched from the replaced right hepatic artery in two cases. Two IPDAs were observed in the remaining eight cases: one from the J1A and the other from the SMA (n = 5); and both from the J1A (n = 3). The IPDA(s) bifurcated from the more cranial side than the MCA and the DJUV intersecting point in all cases.

### 3.4 Distance between the MCA bifurcation and the DJUV intersecting point

**FIGURE 5:**
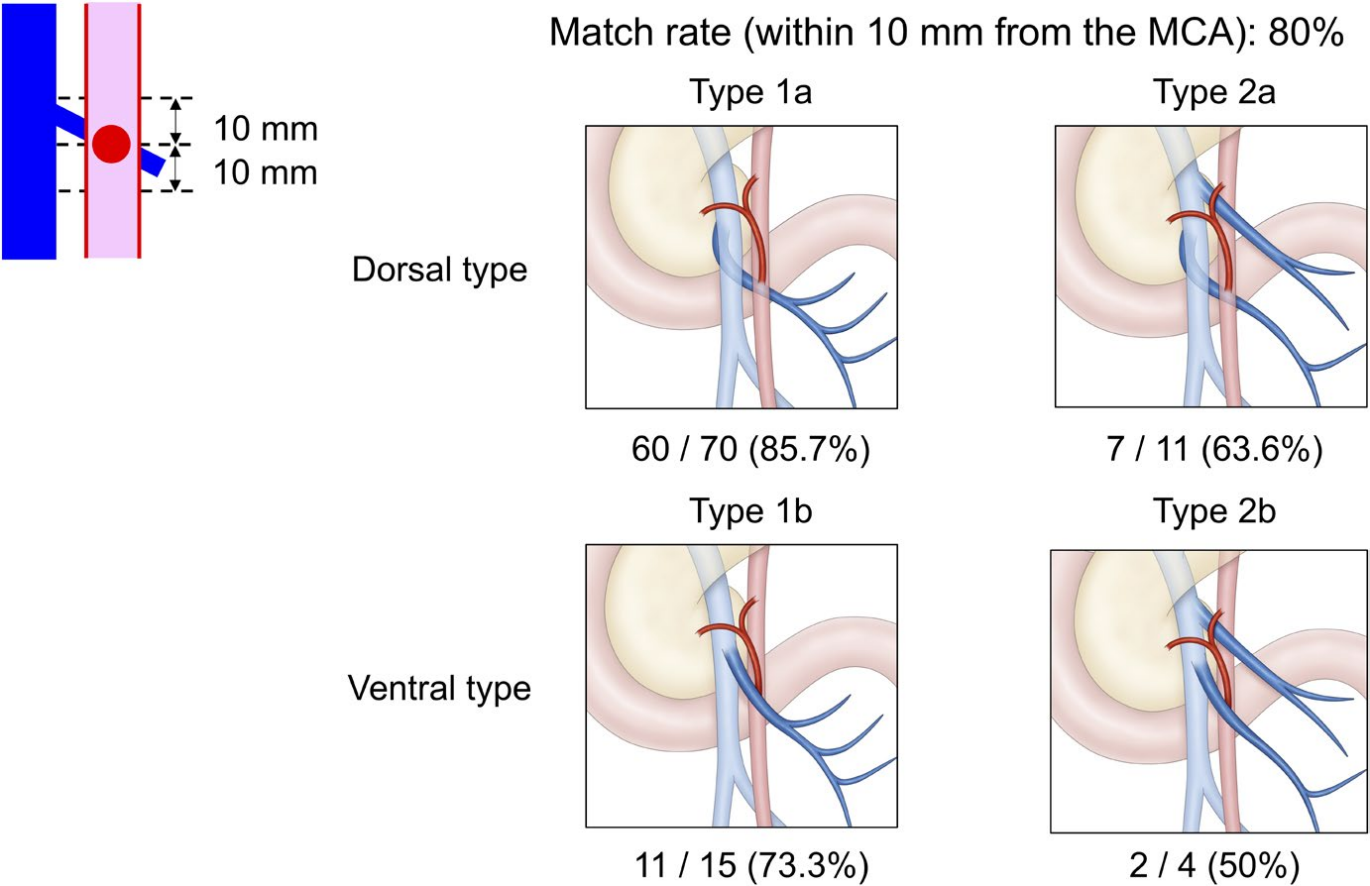
Positional relationship of the MCA and the DJUV. Matching was defined as the distance between the bifurcation of the MCA and the intersection of the DJUV and SMA (DJUV crossing point) within 10 mm. Type 1a had a match rate of 85.7%. Type 1b had a match rate of 73.3%. The match rates of Type 2a and Type 2b were lower, at 63.6% and 50%, respectively. The match rate for all cases was 80%. MCA, middle colic artery; DJUV, duodenojejunal uncinate process vein

According to the General Rules for the Study of Pancreatic Cancer, published by the Japan Pancreas Society, the lymph nodes located between the bifurcation of the MCA and the root of the SMA are regional in cases of cancer of the head of the pancreas (Figure 5).^18^ If the distance between the bifurcation of the MCA and the intersection of the DJUV and SMA (DJUV intersecting point) is short; dissecting along the DJUV will reach the SMA at around the bifurcation of the MCA, which is at the distal end of the regional lymph nodes. A match was defined as a distance of within 10 mm. When the DJUV was equivalent to the J1V (Type 1), the matching rate was 85.7% for the dorsal type and 73.3% for the ventral type. When the DJUV was equivalent to the J2V (Type 2), the rates were 63.6% and 50%, respectively. The matching rate for all cases was 80%. Of the 20 nonmatching cases, the DJUV crossed the SMA 11 mm or more cephalad to the MCA bifurcation in 10 cases.

### 3.5 Number of jejunal arteries branching between the root of the SMA and the DJUV intersecting point

A single jejunal artery (J1A) was identified in nine cases (Table 1). Two arteries (J1A and J2A) were detected in 48 cases, three arteries (J1A, J2A, J3A) in 34 cases, and four arteries (J1A, J2A, J3A, and J4A) in nine cases.

**TABLE 1:** Numbers of cases in which jejunal arteries branched between the root of the SMA and the DJUV intersecting point

Jejunal artery branch	Number of cases
J1A	9
J1A + J2A	48
J1A + J2A + J3A	34
J1A + J2A + J3A + J4A	9

## 4 DISCUSSION

In 1982, Heald et al described the concept of the mesorectum, the importance of the surgical principle of the “holy plane,” and total mesorectal excision during rectal cancer surgery.^19^ In 2007, inspired by the concept of the mesorectum, Gockel et al advocated the concept of the mesopancreas.^6^ In Japan, this region is defined as the “pancreatic head nerve plexus II” in the General Rules for the Study of Pancreatic Cancer by the Japan Pancreas Society.^18^ Kawabata et al advocated the “mesopancreatoduodenum” as an advance of the mesopancreas concept, which expanded the area for lymphadenectomy to the left side of the SMA.^10^, ^12^ Although several surgical procedures have been proposed for mesopancreas or mesopancreatoduodenum resection, the caudal borders, particularly the jejunal mesenteric dissecting line, vary in each report.^10^, ^11^, ^14^, ^16^ The present study is the first to determine the anatomical relationships between DJUV and key blood vessels associated with PD, to evaluate mesopancreatoduodenum resection guided by DJUV.

In this study the J1V was regarded as the DJUV (Type 1) in 85% of cases, and the J2V was regarded as the DJUV in 15% of cases. The J1V ran on the dorsal side of the SMA in 70 cases (84%) in which the J1V corresponded to the DUJV, and the J2V ran on the dorsal side of the SMA in 11 cases (73%) in which the J2V corresponded to the DUJV. Previous studies have reported that the J1V runs dorsal to the SMA in 63%–91% of cases.^15^, ^16^, ^20^, ^21^ Nagakawa et al reported that the J1V was connected to the uncinate process in 31 of 41 cases (73.8%), and the J2V was connected to the uncinate process in 10 of 41 cases (23.8%) in laparoscopic PD.^22^

Few studies have analyzed the anatomical relationships between the jejunal veins and the IPDA and the IPDV, which are included in the mesopancreatoduodenum.^16^ In the present results, three IPDV drainage patterns were observed (Figure 3). In all cases, the IPDVs joined the DJUV or the more cranial side of the SMV. This implies that it is possible to safely dissect between the uncinate process and the SMV by rotating the SMV‐DJUV confluence and dissecting along the DJUV, despite the fact that manipulation of this region is associated with a high risk of bleeding in PD patients.^15^

As shown in Figure 4, there were several variations in the branching pattern of the IPDA. The IPDA arose from J1A or J2A in most cases, and all branches were located on the cranial side of the DJUV. This means that all of the IPDA(s) could easily be identified and divided by dissecting the area around the SMA, starting from the intersecting point of the DJUV toward the root of the SMA. Therefore, this approach is thought to be effective in curative resection of the mesopancreatoduodenum, including all the IPDA(s) and nerve plexuses around the IPDA(s).

The positional relationship between the DJUV and the bifurcation of the MCA matched (within 10 mm) in 80% of cases. The matching rate was 85.7% for the most frequent type (Type 1a). These results indicate that dissection along the DJUV will reach close to the vicinity of the root of the MCA in most cases. In Japan, the lymph nodes located between the bifurcation of the MCA and the root of the SMA are considered regional lymph nodes, and lymphadenectomy in this area around the SMA is required during radical PD for cancer of the pancreatic head. The frequency of lymph node metastasis in this area has been reported to be 11%–40%.^23^, ^24^, ^25^, ^26^, ^27^ Dissection around the SMA from the intersecting point of the DJUV toward the cranial direction facilitates complete resection of these regional lymph nodes. However, the matching rate was lower in Type 2 cases; such cases require special attention if regional lymphadenectomy is planned.

As shown in Table 1, many jejunal arteries ramify between the root of the SMA and the bifurcation of the MCA, so there is often confusion as to which artery is being dissected when mesenteric resection is performed along the jejunal artery. On the other hand, the DJUV is easy to confirm in the mesentery, so it can be considered a more useful anatomical landmark than the jejunal artery for the standard surgery.

If pancreatic cancer has invaded the confluence of the DJUVs, the confluence must be resected to achieve no residual tumor (ie, R0). However, Kobayashi et al reported that 1 of 32 patients who underwent DJUV sacrifice exhibited severe congestion of the jejunal limb, requiring an emergency jejunal resection.^20^ In this study, the DJUV (J1V) had one to five jejunal veins entering the DJUV in most typical Type 1a cases, and the J1V and J2V were nearly confluent in 28 of 70 Type 1a cases (40%) (data not shown).^20^ If pancreatic cancer has invaded the DJUV, it is necessary to consider reconstruction based on the anatomical findings, including peripheral arcades. Even in such cases, DJUV‐guided mesenteric resection is also effective when considering vascular reconstruction.

**FIGURE 6:**
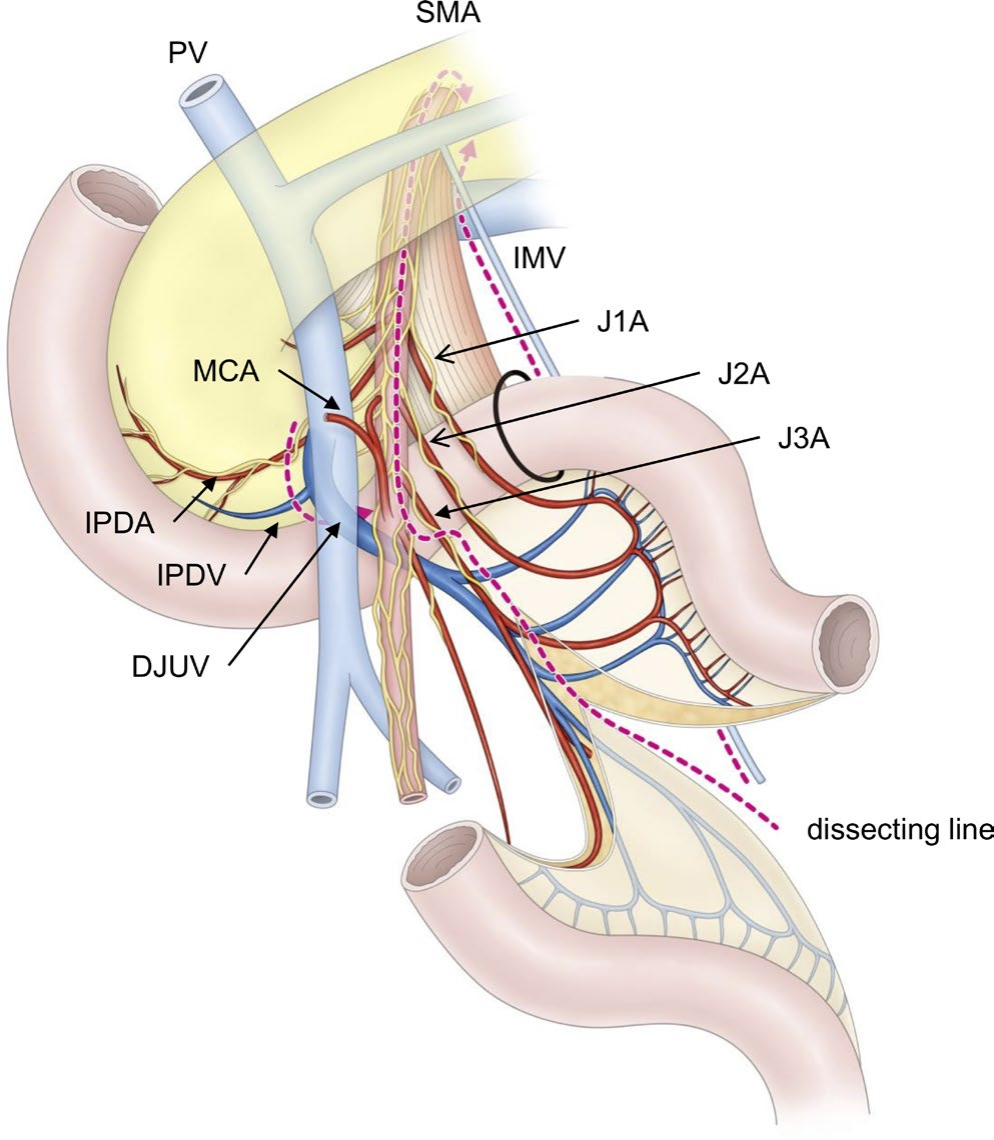
Surgical procedures for resecting the mesopancreatoduodenum using the DJUV as a landmark in Type 1a with typical anatomy. Sequentially dissecting the veins from the region of the SMV‐DJUV confluence, while inverting the SMV. The DJUV bifurcation was confirmed behind the SMV and serves as a landmark of the endpoint during mesopancreatoduodenum resection. In addition, resecting the caudal border of the mesopancreatoduodenum using the JDUPV as a guide from the left side

Although these anatomical advantages imply the utility of the DJUV as a landmark for mesopancreatoduodenum resection, there are some drawbacks regarding the jejunal arteries. In this study, only the J1A or J1A+J2A originated from the SMA between its root and the DJUV intersecting point in 57% of cases. However, in the other 43% of cases, the J3A or J3A+J4A branched between the two points (Table 1). In such cases, if the duodenojejunal mesentery is transected along the DJUV, multiple jejunal arteries, such as the J3A and J4A, must be transected, which may cause ischemic damage to the elevated jejunum during PD. In clinical practice, circulation from the arterial arcade occasionally compensates for the blood supply, but this is not evaluated preoperatively. Therefore, it may be necessary to shift the cut line of the mesojejunum to the more cranial side in cases in which the jejunal arteries override the DJUV and are distributed along the periphery of the jejunal mesentery (ie, along the J2A) (Figure 6).

This study had some limitations. MD‐CT may not reveal all branches of the SMV and SMA, especially the IPDV. Moreover, its retrospective design may be associated with bias. Another is the relatively small sample size. As the vessel anatomy around the head of the pancreas varies widely, further investigation of a larger number of cases is needed to assess the anatomy of the DJUV as a landmark for mesopancreatoduodenum resection. Furthermore, surgical outcomes and patients' prognoses should be evaluated to confirm the validity of the DJUV‐guided method.

In conclusion, based on an analysis of the vascular anatomy and positional relationships between the DJUV and key vessels, this study implies the possibility of using the DJUV as a landmark for mesopancreatoduodenum resection. Dissecting along the DJUV as a caudal border may be useful for safe and oncologically curative PD. We believe that this concept contributes to the establishment of a standard PD procedure.

## DISCLOSURE

Conflict of Interest: The authors declare no conflicts of interest for this article.

Funding: No funding was received for this study.

Ethics: All procedures performed in studies involving human participants were in accordance with the ethical standards of the institution and research committee and with the 1964 Helsinki Declaration and its later amendments or comparable ethical standards. This study was approved by the Ethics Review Boards of the Ehime University School of Medicine and Ehime Central Hospital. Informed consent was by the opt‐out principle and is disclosed on the study website (https://www.m.ehime‐u.ac.jp/school/surgery1/wp‐content/uploads/2020/10/4bd601621bf36c0ac5416707937c91d1.pdf), which included general information and provided an opportunity to decline to participate in this study.

Author contributions: Tohyama and Honjo contributed to the study conception and design. Tohyama, Honjo, Tamura, Takai, Sakamoto, Ogawa, Watanabe, Ohtani, and Takada participated in data collection or management. Tohyama, Honjo, and Takada contributed to the data analysis and interpretation of the data. Tohyama and Honjo drafted the article. Tohyama, Honjo, and Takada contributed to the article writing and editing. Tohyama and Takada provided critical review. All authors read and approved the final article.
